# Parental knowledge, attitudes and perception of pneumococcal disease and pneumococcal conjugate vaccines in Singapore: a questionnaire-based assessment

**DOI:** 10.1186/s12889-016-3597-5

**Published:** 2016-09-02

**Authors:** Choon How How, Priscilla Phua See Chun, Fakrudeen Shafi, Rupert W. Jakes

**Affiliations:** 1 Changi General Hospital, Singapore, Singapore; 2 Singhealth Polyclinics, Singapore, Singapore; 3 GSK Vaccines, Singapore, Singapore; 4 Present: Abbott Laboratories Pte Ltd, Singapore, Singapore; 5 GSK Pharmaceuticals Limited, Bangalore, India

**Keywords:** Survey, Invasive pneumococcal disease, Pneumococcal conjugate vaccine, Cost

## Abstract

**Background:**

Under the National Childhood Immunisation Schedule (NCIS) in Singapore most vaccines are provided free while some, including pneumococcal conjugate vaccines (PCV), added to the NCIS in October 2009, are not free. In contrast to ≥95 % coverage achieved for recommended childhood vaccines that are free, 2013 coverage of the PCV booster dose was 58.9 % (for unclear reasons). To date, no population impact on pneumococcal disease (PD) has been observed. We conducted a questionnaire-based study of parents of young children to assess the value of PCV to parents, and to quantify the extent to which vaccine cost is a barrier to PCV uptake in Singapore.

**Methods:**

A single, trained interviewer administered a questionnaire to 200 parents ≥21 years of age with young children attending the Singapore Sengkang Polyclinic. The questionnaire asked closed-ended questions on parents’ knowledge about PD and PCV. A 5-point Likert scale measured perceived benefits and barriers to PCV vaccination.

**Results:**

There were 162 parents whose children were either PCV-vaccinated or who intended to vaccinate their child with PCV (Vaccinated group), and 38 whose children were non-PCV vaccinated or who did not intend to vaccinate (Unvaccinated group). The odds ratio for PCV vaccination among parents who perceived cost as a barrier was 0.16 (95%CI 0.02–1.23). Compared to the Vaccinated group, parents in the Unvaccinated group were less willing to pay for PCV (50.0 %/94.4 %). Compared to the Vaccinated group, fewer parents in the Unvaccinated group had heard about PD (34.2 %/82.1 %) or PCV (36.8 %/69.1 %), or perceived that PD was a threat to their child. Fewer parents in the Unvaccinated group knew that vaccination could prevent PD (28.9 %/77.8 %), or reported that PCV vaccination was recommended to them by any source (63.2 % had no PCV recommendation, versus 20.4 %). When informed that PCV is included in the NCIS only 65.8 % of parents in the Unvaccinated group, versus 98.8 % in the Vaccinated group, indicated that they would be willing to vaccinate their child.

**Conclusions:**

Cost considerations, not having vaccination recommended to parents and a lack of knowledge among parents of the benefits of PCV to the child may adversely impact PCV uptake in Singapore.

**Electronic supplementary material:**

The online version of this article (doi:10.1186/s12889-016-3597-5) contains supplementary material, which is available to authorized users.

## Background

*Streptococcus pneumoniae* is one of the major causes of bacterial meningitis, sepsis and respiratory infection in children [1]. The World Health Organization (WHO) estimates that 476,000 deaths in children <5 years of age in 2008 were caused by pneumococcal infection [2]. Currently licensed pneumococcal conjugate vaccines (PCVs) have proven to be highly effective in preventing morbidity and mortality due to Invasive Pneumococcal Disease (IPD), pneumonia and otitis media in children, with evidence of herd protection in unvaccinated age groups [3, 4].

Prior to the availability of PCVs in Singapore (2005), the annual incidence of hospitalised “pneumococcal disease” (defined using the International Classification of Disease ICD-9 codes that included pneumococcal pneumonia, meningitis, peritonitis, septicaemia and a less specific code for pneumococcal bacterial infection in unspecified sites) among children <5 years of age was estimated to be 38.4/100,000 population (1997–2004) [5]. The annual incidence in Singapore was similar to the overall incidence of IPD in Europe prior to PCV implementation (31.1/100,000: estimated from multiple studies of diverse design among cohorts aged ≤6 years between 1980 and 2005) [6]. By contrast, using a more stringent case definition of positive cultures collected from children aged <5 years admitted to the KK Women’s and Children’s Hospital, the largest children’s hospital in Singapore, (1998–2004), the annual incidence of laboratory-confirmed IPD was 13.3/100,000 population [7]. The annual incidence in children aged <2 years was 16.9/100,000 population [7]. In another study the annual incidence of IPD requiring hospitalisation among <5 year olds in Hong Kong was 15.6/100,000 from 1995 to 2004 and 16.1/100,000 from 2000 to 2005) [8].

Compliance with the National Childhood Immunisation Schedule in Singapore is generally very high, with at least 95 % coverage of recommended vaccines that are provided free to families; for example, 2012 coverage of the third dose of hepatitis B and poliovirus vaccines was 97 % [9]. The 7-valent PCV (PCV7) was licensed in Singapore in 2005 and was recommended in the National Childhood Immunisation Schedule from October 2009, but at a cost to families [10]. PCV in Singapore is administered as a 2-dose primary immunisation course at 3 and 5 months of age, with a booster administered at 12 months of age. In 2013 coverage of two doses of PCV in Singapore by age 1 year was 79.6 %, and coverage of the booster dose by age 2 years was 58.9 % [11]. PCV vaccination has been associated with a decrease in paediatric IPD due to vaccine-serotypes in Singapore (PCV7 and PCV13 after 2011), and a commensurate increase in IPD due to non-vaccine types, with no change in overall IPD notifications in children since the introduction of PCV [12–14].

Thus, although PCV has been included in the NCIS in Singapore for six years, coverage remains low, and a population impact on IPD has not been observed [12]. Assuming the total cost of the recommended 3-dose schedule in Singapore is $500, the cost of one dose is approximately $167 [15]. The average household monthly income per household member in 2014 was $2380 [16]. Therefore, the cost of one dose represents 7 % of the average household monthly income per household member. Singapore’s gross domestic product per capita is one of the highest in the world [17]. In a survey of mothers of young children in Japan, another high income country, low coverage of voluntary vaccines (rotavirus, mumps, varicella and hepatitis B) was primarily attributed to vaccine cost [18]. In a study conducted in Indonesia, a low-middle income country, vaccine cost and lack of knowledge about the severity of the targeted disease were important factors that influenced PCV uptake [19].

We conducted a questionnaire-based study of parents, the primary decision makers and payers of elective vaccinations on the national schedule, of young children in order to assess the value of PCV to parents, and to quantify the extent to which the cost of the vaccine is a barrier to the uptake of PCV in Singapore. We also explored other factors that may influence PCV uptake in Singapore.

## Methods

### Study design

This was an observational, cross-sectional interviewer-administered survey conducted at the SingHealth Polyclinic in Sengkang, Singapore between 14 May 2014 and 09 July 2014. SingHealth is one of two public health providers and provides primary care (including immunisation) in the central east and north-east regions of Singapore. SingHealth encompasses a network of nine polyclinics; the Sengkang polyclinic was selected as the study site because it serves the fastest growing new residential estate in Singapore, with a dense concentration of young families [20]. The distribution of young children resident across Singapore is heterogeneous [21]. Together, Sengkang and the adjacent region of Punggol form the most concentrated population of children less than 5 years of age in Singapore [21]. Conducting the study in the most concentrated population of subjects of interest is likely to be representative of parents and children across Singapore.

Eligible parents were ≥21 years of age, had at least one child born on or after 01 November 2009, and had brought their child to the vaccination clinic at the SingHealth Polyclinic in Sengkang. Individuals could only complete the survey once.

The study was approved on 07 February 2014 by the SingHealth Centralised Institutional Review Board (Ref: 2014/116/E) and conducted in accordance with the Declaration of Helsinki and ‘good clinical practice’ principles. All participants gave written informed consent before enrolment. All data collected were anonymous.

### Survey methods and data collection

The study questionnaire assessed the knowledge and attitudes of parents across three broad domains: knowledge about pneumococcal disease, knowledge about PCV, and the perceived benefits and barriers of vaccination with PCV. The questionnaire was pilot-tested in 10 volunteers prior to study start to assess comprehension and to determine the time needed to conduct the interviews. The questionnaire was translated into the major languages of Singapore by a professional translator. The translations were submitted to the SingHealth Centralised Institutional Review Board.

All questionnaires were administered by a single, trained, interviewer who was not a member of the Polyclinic staff and not involved in administering childhood vaccinations. After explaining the study and enrolling the subject, the interviewer recorded basic demographic information and socioeconomic status. The interviewer asked participants for their responses to a series of closed-ended questions under the ‘knowledge about pneumococcal disease’ and ‘knowledge about PCV’ domains. Parent’s knowledge about the risk, cause, severity and clinical manifestations of pneumococcal disease and how it can be prevented was assessed. Parents were questioned about PCV, including its availability on the National Childhood Immunisation Schedule and information sources and factors influencing their decision to vaccinate. Benefits, such as the ability of the vaccine to protect, and barriers to vaccination including the perception of harms, costs, and issues of vaccine access, were recorded on a 5-point Likert scale. Each interview lasted approximately 20–30 min. The full list of questions and responses is provided in the Supplement (Additional file 1: Table S1, Additional file 2: Table S2, Additional file 3: Table S3, Additional file 4: Table S4).

Subjects were approached using a systematic sampling frame. Appointment numbers for pre-booked and ‘walk-in’ patients were randomly mixed according to time of arrival to the clinic, or time of transfer from other clinic service stations to the vaccination queue list. A sampling frame of one in five numbers was applied to the queue display pad in the vaccination service room; that is, the first, sixth, 11^th^ and so on. The queue numbers in those positions were called out in the waiting area and the parents holding these identified queue numbers were then invited for screening for eligibility.

### Statistical analysis

There were two study groups: those parents whose children were either PCV-vaccinated or who intended to vaccinate their child with PCV (Vaccinated group), and those parents whose children were not vaccinated with PCV, or who did not intend to vaccinate their child with PCV (Unvaccinated group).

We used a logistic regression model to quantify the extent to which vaccine cost and the other survey domains influenced uptake. Odds ratios (OR) with 95 % confidence intervals (CI) were calculated for PCV vaccination (dependent variable) based on whether cost (independent variable) was indicated as being too high. Ordinal outcomes (5-point Likert scale information related to the question ‘*the cost of the PCV is too high*’) were converted into a binary response, with values below the “neutral” response considered as “No” and values above the “neutral” response considered as “Yes” for barriers in terms of cost. Those subjects who answered “neutral” to the independent variable were not included in the logistic regression analysis related to the primary endpoint.

Sensitivity analyses using the 5-point Likert scale information as continuous independent variables (considering the score of 1 to 5) were also conducted using a logistic regression model.

Descriptive summary statistics (frequencies and percentages) for all domains, as well as the polychoric correlation co-efficient between the 15 items measured on the 5-point Likert scale related to the perceived benefits and barriers to vaccination domain, was computed. Exploratory post-hoc analyses compared groups in terms of their demographic characteristics, parental attitudes and intentions concerning PCV based on cost factors, and parent’s knowledge about pneumococcal disease and PCV, using the Fisher’s exact test or Mann-Whitney-Wilcoxon test.

In view of the sample size, the number of items related to perceived benefits and barriers to vaccination and the expected correlation among these items, an exploratory factor analysis using polychoric correlation co-efficient was performed. The factor analysis reduced the large number of variables of interest to a smaller group of unobservable (latent) factors. This was followed by a multiple logistic regression model that considered vaccination status as the dependent variable and the identified latent factors as independent variables [22]. A *p*-value <0.05 was used to indicate that a statistically significant difference might exist between groups.

### Sample size

The sample size was estimated based on the assumed theoretical proportions of affirmative responses in terms of cost being a barrier to the uptake of PCV. We assumed that PCV vaccine coverage in Singapore might be around 50 % at the time of the study, and that cost might be a barrier to vaccination in 25 % of families. Considering an even distribution of responses across the 5-point Likert scale to the question’t*he cost of PCV is too high*’, and that 20 % of parents would respond ‘Neutral’ to this statement, the study had 81 % power to detect an OR of four in the proportion of affirmative responses between Vaccinated and Unvaccinated groups (or an OR of 0.25 in the Unvaccinated compared to the Vaccinated group), when there were 80 subjects in each group (Total enrolled cohort of 200).

The statistical analyses were performed using SAS® version 9.2 and SAS® Drug and Development version 3.5. We used PASS version 2005 software for sample size calculations.

## Results

### Characteristics of the study population

There were 200 parents enrolled and all contributed data to the final analysis. There were 162 parents in the Vaccinated group and 38 parents in the Unvaccinated group. The median age of the child at interview was 6 months (range 0–27 months) in the Vaccinated group and 4 months (range 0–19 months) in the Unvaccinated group. The interviewees were mothers for 68.5 and 57.9 % of children in the respected groups. Demographic features of the Vaccinated and Unvaccinated parents appeared to be similar (Table 1) (*p* > 0.05 for all characteristics). Children in the Unvaccinated group appeared to be younger than children in the Vaccinated group (*p* < 0.01).

**Table 1:** Demographic characteristics of interviewed parents according to PCV vaccination status of their child

Characteristics	Categories	Vaccinated group	Unvaccinated group
		*N* = 162	*N* = 38
Age of the child at interview in months	Mean (SD)	7.3 (5.6)	4.8 (5.1)
	Median (range)	6 (0–27)	4 (0–19)
Age category of the interviewed parent, *n* (%)	21–30 years	59 (36.4)	15 (39.5)
	31–40 years	97 (59.9)	18 (47.4)
	≥41 years	6 (3.7)	5 (13.2)
Gender of the interviewed parent, *n* (%)	Male	51 (31.5)	16 (42.1)
	Female	111 (68.5)	22 (57.9)
Ethnicity, *n* (%)	Chinese	86 (53.1)	19 (50.0)
	Indian	8 (4.9)	3 (7.9)
	Malay	62 (38.3)	15 (39.5)
	Other	6 (3.7)	1 (2.6)
Residency status, *n* (%)	Permanent Resident	10 (6.2)	2 (5.3)
	Singapore citizen	149 (92.0)	36 (94.7)
	Other	3 (1.9)	0 (−)
Highest education of the interviewed parent, *n* (%)	Primary	2 (1.2)	2 (5.3)
	Secondary	30 (18.5)	8 (21.1)
	Post-secondary	130 (80.2)	28 (73.7)
Combined monthly household income, *n* (%)	<1000 SGD	4 (2.5)	1 (2.6)
	1001 to 3000 SGD	36 (22.2)	12 (31.6)
	3001 to 5000 SGD	54 (33.3)	11 (28.9)
	>5000 SGD	58 (35.8)	11 (28.9)
	Prefer not to answer	10 (6.2)	3 (7.9)

*Vaccinated group* Parents whose children had received PCV or parents who intended to have their child vaccinated, *Unvaccinated group* Parents whose children had not received PCV or parents who had no intention of having their child vaccinated, *N* total number of parents, *n (%)* number (percentage) of parents in a given category, *PCV* pneumococcal conjugate vaccine, *SD* standard deviation, *SGD* Singapore dollars

### Cost as a barrier to PCV vaccination

There were 63.0 % (126/200) of parents overall (61.1 % [99/162] of parents in the Vaccinated group and 71.1 % [27/38] in the Unvaccinated group) who answered ‘Moderately Agree’ or ‘Strongly Agree’ to the question ‘*The cost of PCV is too high*’ (Table 2). The OR for PCV vaccination with cost as a barrier was 0.16 (95 % CI 0.02–1.23): that is, the odds (or likelihood) of letting their child be vaccinated with PCV was 0.16 in the group of parents who believed that cost is a barrier, when compared to the parents who did not believe that cost is a barrier.

**Table 2:** Parental attitudes and intentions concerning PCV based on cost factors^a^

Question	Response	Vaccinated group *N* = 162 *n* (%)	Unvaccinated group *N* = 38 *n* (%)	p-value
The cost of PCV is too high (5-Point Likert scale)^b^	Strongly Disagree	7 (4.3)	0 (−)	0.21
	Moderately Disagree	16 (9.9)	1 (2.6)	
	Neutral	40 (24.7)	10 (26.3)	
	Moderately Agree	49 (30.2)	12 (31.6)	
	Strongly Agree	50 (30.9)	15 (39.5)	
If the PCV was free of charge, would you be willing for your child to be vaccinated with the vaccine?	No	1 (0.6)	9 (23.7)	<0.01
	Yes	161 (99.4)	29 (76.3)	
If you had to pay for the PCV, would you be willing for your child to be vaccinated with the vaccine?	No	9 (5.6)	19 (50.0)	<0.01
	Yes	153 (94.4)	19 (50.0)	
How much are you willing to pay for your child to be vaccinated with the PCV?	<50 SGD per jab	73 (47.7)	13 (68.4)	
	50 to 100 SGD per jab	55 (35.9)	6 (31.6)	0.14
	101 to 150 SGD per jab	25 (16.3)	0 (−)	
Do you intend to let your child receive the PCV?	Yes	76 (46.9)	0 (−)	-
	No	0 (−)	38 (100)	
	Already vaccinated	86 (53.1)	0 (−)	

*Vaccinated group* Parents whose children had received PCV or parents who intended to have their child vaccinated, *Unvaccinated group* Parents whose children had not received PCV or parents who had no intention of having their child vaccinated, *N* total number of parents, *n (%)* number (percentage) of parents in a given category, *PCV* pneumococcal conjugate vaccine, *SGD* Singapore dollars

*p*-values using Fisher's exact test

^a^Subset of questions and responses. Responses to all questions are provided in full in the Supplement

^b^The odds ratio for PCV vaccination among parents who perceived cost as a barrier was 0.16 (95 % CI 0.02–1.23)

In sensitivity analysis after the 5-point Likert scale was treated as a continuous variable, (using the scores from 1 to 5 for strongly disagree to strongly agree), cost remained a barrier (OR 0.73).

Compared to parents in the Vaccinated group, parents in the Unvaccinated group were less willing to pay for PCV vaccination (94.4 % Vaccinated, 50.0 % Unvaccinated) (Table 2). For those that were willing to pay, 16.3 % in the Vaccinated group were willing to pay 101 to 150 SGD (Singapore Dollar) per dose, versus 0 % in the Unvaccinated group (Table 2). Parents in the Unvaccinated group were also less willing to vaccinate with PCV if the vaccine was free (76.3 % Unvaccinated, 99.4 % Vaccinated), suggesting that cost was not the only factor affecting uptake. Of the 9 parents who were unwilling to vaccinate if PCV was free, 3 did not know about pneumococcal disease and one was not aware of PCV.

### Knowledge and attitudes as barrier to PCV vaccination

Compared to the Vaccinated group, fewer parents in the Unvaccinated group had heard about pneumococcal disease (82.1 % Vaccinated, 34.2 % Unvaccinated) or PCV (69.1 % Vaccinated, 36.8 % Unvaccinated) (Table 3). There were fewer parents in the Unvaccinated group than the Vaccinated group who knew that babies were at risk of pneumococcal disease (61.5 % Unvaccinated, 81.2 % Vaccinated), or who perceived that pneumococcal disease was a threat to their child. Fewer parents in the Unvaccinated group knew that vaccination could prevent pneumococcal disease (84.6 % Unvaccinated, 94.7 % Vaccinated), or had PCV vaccination recommended to them by any source (63.2 % in the Unvaccinated group had no PCV recommendation versus 20.4 % in the Vaccinated group). Information from healthcare professionals was the most influential source of information for both groups. Twice the proportion of parents in the Unvaccinated group versus the Vaccinated group indicated that the internet was the most influential source of information (34.2 % Unvaccinated, 13.6 % Vaccinated). Knowing that PCV is included in the National Childhood Immunisation Schedule in Singapore, only 65.8 % of parents in the Unvaccinated group, versus 98.8 % in the Vaccinated group, indicated that they would be willing for their child to be vaccinated.

**Table 3:** Parental knowledge concerning pneumococcal disease and PCV^a^

		Vaccinated group *N* = 162 *n* (%)	Unvaccinated group *N* = 38 *n* (%)	*p*-value
Have you heard about pneumococcal disease?	Yes	133 (82.1)	13 (34.2)	<0.01
	No	29 (17.9)	25 (65.8)	
Are babies at risk of developing pneumococcal disease?^b^	Yes	108 (81.2)	8 (61.5)	0.13
	No	7 (5.3)	1 (7.7)	
	Do not know	18 (13.5)	4 (30.8)	
Can vaccination prevent pneumococcal disease?^b^	Yes	126 (94.7)	11 (84.6)	0.18
	No	7 (5.3)	2 (15.4)	
	Do not know	29 (21.8)	2 (15.4)	
The chance of your child catching pneumococcal disease is;^b^	Very likely	7 (5.3)	0 (−)	0.19
	Moderately likely	36 (27.1)	1 (7.7)	
	Neither likely nor unlikely	58 (43.6)	9 (69.2)	
	Moderately unlikely	24 (18.0)	2 (15.4)	
	Very unlikely	8 (6.0)	1 (7.7)	
The consequences of pneumococcal disease for a child are;^b^	Very severe	68 (51.1)	4 (30.8)	0.08
	Moderately severe	41 (30.8)	4 (30.8)	
	Neither severe or mild	21 (15.8)	3 (23.1)	
	Moderately mild	0 (−)	1 (7.7)	
	Very mild	3 (2.3)	1 (7.7)	
Have you heard about PCV before?	Yes	112 (69.1)	14 (36.8)	<0.01
	No	50 (30.9)	24 (63.2)	
Is PCV included in the NIP in Singapore?	Yes	92 (56.8)	13 (34.2)	0.01
	No	29 (17.9)	6 (15.8)	
	Do not know	41 (25.3)	19 (50.0)	
PCV does more good than harm	Strongly Disagree	1 (0.6)	0 (−)	<0.01
	Moderately Disagree	1 (0.6)	1 (2.6)	
	Neutral	25 (15.4)	21 (55.3)	
	Moderately Agree	68 (42.0)	14 (36.8)	
	Strongly Agree	67 (41.4)	2 (5.3)	
The PCV vaccine works	Neutral	95 (58.6)	32 (84.2)	<0.01
	Moderately Agree	45 (27.8)	6 (15.8)	
	Strongly Agree	22 (13.6)	0 (−)	
There are too many doses required for vaccination with the PCV	Strongly Disagree	6 (3.7)	1 (2.6)	0.95
	Moderately Disagree	11 (6.8)	2 (5.3)	
	Neutral	54 (33.3)	11 (28.9)	
	Moderately Agree	63 (38.9)	18 (47.4)	
	Strongly Agree	28 (17.3)	6 (15.8)	
My child does not need the PCV as he/she is strong enough to cope with pneumococcal disease	Strongly Disagree	49 (30.2)	1 (2.6)	<0.01
	Moderately Disagree	67 (41.4)	7 (18.4)	
	Neutral	43 (26.5)	26 (68.4)	
	Moderately Agree	2 (1.2)	4 (10.5)	
	Strongly Agree	1 (0.6)	0 (−)	
There are too many childhood vaccinations for my child to take	Strongly Disagree	9 (5.6)	2 (5.3)	0.05
	Moderately Disagree	20 (12.3)	0 (−)	
	Neutral	39 (24.1)	7 (18.4)	
	Moderately Agree	63 (38.9)	15 (39.5)	
	Strongly Agree	31 (19.1)	14 (36.8)	
Who recommended that your child receive the PCV^c^	Doctor	51 (31.5)	6 (15.8)	–
	Nurse	88 (54.3)	9 (23.7)	
	Friends or Family	20 (12.3)	2 (5.3)	
	Child care/day care	2 (1.2)	0 (−)	
	No one recommended	33 (20.4)	24 (63.2)	
Which of the following sources of information would you consider most influential to your decision making regarding vaccination with PCV^c^?	Healthcare Professional	151 (93.2)	33 (86.8)	–
	Media: TV, radio, newspapers, magazines	40 (24.7)	9 (23.7)	
	Friend, family members	51 (31.5)	13 (34.2)	
	Brochures, leaflets, posters in doctor clinics	78 (48.1)	19 (50.0)	
	The internet	22 (13.6)	13 (34.2)	
	Other	3 (1.9)	0 (-)	
Knowing the PCV is included in the NIP in Singapore. Would you be willing for your child to be vaccinated with the PCV?	Yes	160 (98.8)	25 (65.8)	<0.01
	No	2 (1.2)	13 (34.2)	

*Vaccinated group* Parents whose children had received PCV or parents who intended to have their child vaccinated, *Unvaccinated group* Parents whose children had not received PCV or parents who had no intention of having their child vaccinated, *N* total number of parents, *n (%)* number (percentage) of parents in a given category, *PCV* pneumococcal conjugate vaccine, *SGD* Singapore dollars, *NIP* National Childhood Immunisation Programme

*p*-values using Fisher’s exact test

^a^Subset of questions and responses. Responses to all questions are provided in full in the Supplement

^b^Denominator does not include parents who stated that they did not know about pneumococcal disease

^c^Since a subject can choose more than one option, the percentages will not add up to 100 %

### Perceived benefits and barriers to PCV vaccination

Of 15 questions in the ‘Perceived benefits and barriers to PCV vaccination’ domain, 13 items correlated with at least one other item with a polychoric coefficient value of ≥0.30 (Additional file 5: Table S5), suggesting reasonable factorability; that is, items have a weak to strong positive relationship.

In the factors analysis, communalities for 10 out of 15 items were >0.25, confirming that each item shared some common variance with other items (Table 4). The higher the factor loading (or score), the greater the likely influence of the item on the particular factor. For example, Factor 1 has characteristics very similar to items 1, 8, 9 and 12. The questions related to these items indicate that Factor 1 relates to “perceived benefits” and that similarly, Factor 2 relates to “perceived barriers”. The two-factor solution was preferred as it explained 82.7 % of the overall variance.

**Table 4:** Communality estimates and variance of the items from perceived benefits and barriers domain – Factor analysis^a^

		Factor loadings^b^		
Item (Perceived benefits and barriers)		Factor 1	Factor 2	Communality estimates
1	By allowing my child to take the PCV I will help protect my child from pneumococcal disease	0.82	–	0.68
2	There are too many doses required for vaccination with the PCV	–	0.70	0.50
3	The number of doses of PCV will influence my decision whether to vaccinate my child at a younger or older age	–	0.52	0.27
4	My child does not need the PCV as he/she is strong enough to cope with pneumococcal disease	–0.65	–	0.44
5	PCV causes disease	–0.31	0.38	0.24
6	Vaccination with PCV is too painful for my child	–0.25	0.45	0.27
7	There are too many childhood vaccinations for my child to take	–	0.68	0.50
8	PCV does more good than harm	0.84	–	0.72
9	It is my responsibility to ensure that my child is vaccinated against pneumococcal disease	0.80	–	0.64
10	It is not easy for me to find time to bring my child to the clinic for the PCV	–	0.37	0.14
11	Transportation to the clinic for PCV is a problem	–	0.27	0.10
12	The PCV works	0.53	–	0.28
13	There is no long term benefit of the PCV	−0.24	0.26	0.13
14	There are short-term side effects from the PCV	–	0.32	0.10
15	The cost of the PCV is too high	–	0.52	0.27
	Percentage explained (%)	–	–	82.70

Communality: Proportion of variance in items explained by the factors

Percentage explained: Proportion (Percentage) of variance in the data that is explained by all the factors

*PCV* pneumococcal conjugate vaccine

^a^Factor Analysis (using PROMAX rotation; Unweighted Least Squares [ULS] method; Squared Multiple Correlations [SMC] Priors and MINIMUM EIGEN value >1 to retain the number of factors) was performed to identify the latent factors for perceived benefits and barriers

^b^Factor loadings < 0.2 are suppressed

The OR for Factor 1 from the logistic regression model suggests that the likelihood of parents letting their child vaccinated increases by 5.2 times with each one unit increase in the standardised items related to “perceived benefits” (*p*-value <0.01) (Table 5). Factor 2 suggests the likelihood of parents letting their child vaccinated decreases by 0.75-fold with each one unit increase in the standardized items which are believed to be “barriers” (not statistically significant).

**Table 5:** Estimated coefficients of the fitted logistic regression model for PCV uptake with influencing benefits and barriers as factors

Parameters or Categories	Coefficient	Standard error	*P*-value	Adjusted OR^a^ (95 % CI)
FACTOR 1 (Perceived benefits)	1.69	0.30	<0.001	5.24 (2.89; 9.52)
FACTOR 2 (Perceived barriers)	−0.50	0.31	0.36	0.75 (0.40; 1.39)

*OR* odds ratio, *PCV* pneumococcal conjugate vaccine, *95 % CI of OR* 95 % confidence interval of Odds ratio

^a^the OR for Factor 1 was adjusted for Factor 2, and the OR for Factor 2 was adjusted for Factor 1

## Discussion

Parents make the decision as to whether or not their child receives vaccines. The knowledge, perceived benefits and factors that influence the decision to vaccinate are complex. The decision not to vaccinate has direct implications for the health and well-being of individual children and for the wider community should herd protection effects fail to be achieved due to low coverage in the paediatric population. The uptake of vaccines in the NCIS that are provided free in Singapore is very high [11], which is in marked contrast to that of PCV, which comes at a cost to families, suggesting that cost may be a barrier to vaccination with PCV. We used a questionnaire to explore the perceived benefits and barriers to PCV vaccination in Singapore in order to estimate the value of PCV to parents.

More parents in the Unvaccinated group had not heard about pneumococcal disease or PCV vaccination, and these parents perceived less threat from pneumococcal disease and less benefit from PCV vaccination than parents in the Vaccinated group. Furthermore, more parents in the Unvaccinated group had not had PCV recommended to them by a healthcare professional. These results suggest that efforts and activities to improve parental knowledge about IPD could impact positively on the perceived value of PCV vaccination, which can influence parents’ decisions to vaccinate. An increased role of healthcare providers could potentially impact vaccine uptake, through communicating the risks of IPD and benefits of vaccination to parents directly, and indirectly through provision of informative materials, and via the internet.

Under the ‘perceived benefits and barriers to PCV vaccination’ domain, barriers to vaccination appeared to be the cost of PCV, concerns about PCV side-effects, a perception that there are too many childhood vaccines and too many PCV doses, and issues linked to vaccine access; either because of lack of parental time, or costs associated with transport to the clinic. The administration of combination vaccines has been linked to improved acceptability and coverage rates [23, 24]. Identification of ways to reduce the number of injections required to complete the Singapore vaccination schedule could also improve coverage of PCV, with benefits that could extend to other new vaccines potentially being considered for introduction into the schedule.

This is the first study conducted at a local level to explore the reasons why parents in this community in Singapore do not vaccinate their children with PCV. The study was conducted at a time when the importance of PCV has been recognised by inclusion into the NCIS by policy-makers, but when the benefits of vaccination at a population level have not been optimal. The study questionnaire was refined during a pilot phase and we believe that this improved its internal validity and utility. The exploratory factor analysis and logistic regression indicated that an increase in the perceived benefits of vaccination (i.e., value proposition) is likely to have a greater influence on the decision to vaccinate than attempting to change perceived barriers. The identification of actionable factors provides evidence about which public health measures, health promotion or policy changes could positively influence the perceived value of vaccination.

Our study is potentially limited because the observed percentage of parents who reported ‘neutral’ to the question ‘*the cost of PCV is too high’*, and thus eliminated from the analysis of the primary endpoint, was somewhat higher than anticipated (25 % versus 20 %). Additionally, the distribution of parents between Vaccinated and Unvaccinated groups was not equal (81 % in Vaccinated group and 19 % in the Unvaccinated group), whereas the study was powered according to a 50:50 distribution. Therefore, while the study identified cost as a likely factor influencing parents uptake of PCV, the association was not statistically significant and a definitive conclusion about cost as a barrier cannot be drawn. Finally, our survey was limited to one of the 18 public health centres serving the whole of Singapore, and the conclusions may not be applicable to private clinics.

## Conclusion

Infant vaccination with PCV has been included in the NCIS in Singapore since 2009 but coverage remains sub-optimal. Although changes in the distribution of pneumococcal serotypes have been observed among notified cases of IPD since PCV introduction, the overall number of IPD notifications remains unchanged [12–14]. Our study suggests that cost considerations, as well as lack of knowledge about pneumococcal disease and PCV among parents, reduces the value of vaccination, whatever the perceived benefits. Parents of PCV-unvaccinated children are less likely to have received a recommendation for vaccination. Changes to reduce costs of PCV to families, targeted public health messages to parents and healthcare professionals, and interventions to improve the perception of the value of PCV vaccination have the potential to increase PCV uptake in this community in Singapore.

## Additional files

Additional file 1:
**Table S1.** Perceived benefits and barriers of vaccination as reported by parents. (DOCX 39 kb) Additional file 2:
**Table S2.** Knowledge of the parent about pneumococcal disease. (DOCX 37 kb) Additional file 3:
**Table S3.** Knowledge of parent about pneumococcal conjugate vaccine. (DOCX 36 kb) Additional file 4:
**Table S4.** Parental intent to vaccinate with PCV. (DOCX 37 kb) Additional file 5:
**Table S5.** Polychoric correlation coefficients between items for perceived benefits and barriers of PCV (15 questions ordinal using 5-point Likert scale). (DOCX 38 kb)

## Abbreviations

CI: Confidence interval
IPD: Invasive pneumococcal disease
ICD: International Classification of Disease
OR: Odd ratio
PCV: Pneumococcal conjugate vaccine
PCV7: 7-valent PCV
SGD: Singapore dollar
WHO: World Health Organization
