# Supplementary material for: Parental knowledge, attitudes and perception of pneumococcal disease and pneumococcal conjugate vaccines in Singapore: a questionnaire-based assessment
Source: BMC Public Health. 2016 Sep 2;16(1):923. doi: 10.1186/s12889-016-3597-5 (PMC5010741; doi:10.1186/s12889-016-3597-5)
Supplement: Additional file 1: — Table S1. Perceived benefits and barriers of vaccination as reported by parents. (DOCX 39 kb) [file 12889_2016_3597_MOESM1_ESM.docx]

**Table S1:** Perceived benefits and barriers of vaccination as reported by parents

| **Question** | **Vaccinated group N = 162, n (%)** | | | | | **Unvaccinated group N = 38, n (%)** | | | | |
| --- | --- | --- | --- | --- | --- | --- | --- | --- | --- | --- |
|  | **SD** | **MD** | **N** | **MA** | **SA** | **SD** | **MD** | **N** | **MA** | **SA** |
| 1. By allowing my child to take the PCV, I will help protect my child from pneumococcal disease | 0 | 1 (0.6) | 12 (7.4) | 68 (42.0) | 81 (50.0) | 0 | 0 | 15 (39.5) | 17 (44.7) | 6 (15.8) |
| 2. PCV does more good than harm | 1 (0.6) | 1 (0.6) | 25 (15.4) | 68 (42.0) | 67 (41.4) | 0 | 1 (2.6) | 21 (55.3) | 14 (36.8) | 2 (5.3) |
| 3. It is my responsibility to ensure that my child is vaccinated against pneumococcal disease | 0 | 0 | 11 (6.8) | 48 (29.6) | 103 (63.6) | 0 | 0 | 11 (28.9) | 21 (55.3) | 6 (15.8) |
| 4. It is not easy for me to find time to bring my child to the clinic for the PCV | 44 (27.2) | 37 (22.8) | 38 (23.5) | 33 (20.4) | 10 (6.2) | 6 (15.8) | 7 (18.4) | 17 (44.7) | 7 (18.4) | 1 (2.6) |
| 5. Transportation to the clinic for PCV is a problem | 80 (49.4) | 39 (24.1) | 27 (16.7) | 13 (8.0) | 3 (1.9) | 15 (39.5) | 7 (18.4) | 12 (31.6) | 3 (7.9) | 1 (2.6) |
| 6. The PCV works | 0 | 0 | 95 (58.6) | 45 (27.8) | 22 (13.6) | 0 | 0 | 32 (84.2) | 6 (15.8) | 0 |
| 7. There is no long term benefit of the PCV | 16 (9.9) | 25 (15.4) | 105 (64.8) | 12 (7.4) | 4 (2.5) | 0 | 0 | 35 (92.1) | 3 (7.9) | 0 |
| 8. There are short term side effects from the PCV | 7 (4.3) | 13 (8.0) | 95 (58.6) | 43 (26.5) | 4 (2.5) | 0 | 0 | 30 (78.9) | 8 (21.1) | 0 |
| **9. The cost of the PCV is too high** | **7 (4.3)** | **16 (9.9)** | **40 (24.7)** | **49 (30.2)** | **50 (30.9)** | **0** | **1 (2.6)** | **10 (26.3)** | **12 (31.6)** | **15 (39.5)** |
| 10 .There are too many doses required for vaccination with the PCV | 6 (3.7) | 11 (6.8) | 54 (33.3) | 63 (38.9) | 28 (17.3) | 1 (2.6) | 2 (5.3) | 11 (28.9) | 18 (47.4) | 6 (15.8) |
| 11. The number of doses of PCV will influence my decision whether to vaccinate my child at a younger or older age | 13 (8.0) | 20 (12.3) | 47 (29.0) | 54 (33.3) | 28 (17.3) | 2 (5.3) | 2 (5.3) | 13 (34.2) | 16 (42.1) | 5 (13.2) |
| 12. My child does not need the PCV as he/she is strong enough to cope with pneumococcal disease | 49 (30.2) | 67 (41.4) | 43 (26.5) | 2 (1.2) | 1 (0.6) | 1 (2.6) | 7 (18.4) | 26 (68.4) | 4 (10.5) | 0 |
| 13. PCV causes disease | 29 (17.9) | 39 (24.1) | 85 (52.5) | 8 (4.9) | 1 (0.6) | 3 (7.9) | 8 (21.1) | 24 (63.2) | 3 (7.9) | 0 |
| 14. Vaccination with PCV is too painful for my child | 17 (10.5) | 29 (17.9) | 80 (49.4) | 29 (17.9) | 7 (4.3) | 1 (2.6) | 3 (7.9) | 20 (52.6) | 10 (26.3) | 4 (10.5) |
| 15. There are too many childhood vaccinations for my child to take | 9 (5.6) | 20 (12.3) | 39 (24.1) | 63 (38.9) | 31 (19.1) | 2 (5.3) | 0 | 7 (18.4) | 15 (39.5) | 14 (36.8) |
| **Knowledge of the parent about pneumococcal disease (Questions using Likert scale)** | | | | | | | | | | |
|  | VL | ML | N | MU | VU | VL | ML | N | MU | VU |
| The chance of your child catching pneumococcal disease is | 7 (5.3) | 36 (27.1) | 58 (43.6) | 24 (18.0) | 8 (6.0) | 0 | 1 (7.7) | 9 (69.2) | 2 (15.4) | 1 (7.7) |
|  | VS | MS | N | MM | VM | VS | MS | N | MM | VM |
| The consequences of pneumococcal disease for a child are | 68 (51.1) | 41 (30.8) | 21 (15.8) | 0 | 3 (2.3) | 4 (30.8) | 4 (30.8) | 3 (23.1) | 1 (7.7) | 1 (7.7) |

Vaccinated group = Parents whose children had received PCV or parents who intended to have their child vaccinated, Unvaccinated group = Parents whose children had not received PCV or parents who had no intention of having their child vaccinated, N = total number of parents, n (%) = number (percentage) of parents in a given category, PCV = pneumococcal conjugate vaccine

SD = Strongly Disagree, MD = Moderately Disagree, N=Neutral, MA = Moderately Agree, SA = Strongly Agree.

VL = Very likely, ML = Moderately likely, N = Neither likely nor unlikely, MU = Moderately unlikely, VL = Very unlikely

VS = Very severe, MS = Moderately severe, N = Neither severe or mild, MM = Moderately mild, VM = Very mild
