# Supplementary material for: Parental knowledge, attitudes and perception of pneumococcal disease and pneumococcal conjugate vaccines in Singapore: a questionnaire-based assessment
Source: BMC Public Health. 2016 Sep 2;16(1):923. doi: 10.1186/s12889-016-3597-5 (PMC5010741; doi:10.1186/s12889-016-3597-5)
Supplement: Additional file 2: — Table S2. Knowledge of the parent about pneumococcal disease. (DOCX 37 kb) [file 12889_2016_3597_MOESM2_ESM.docx]

**Table S2**: Knowledge of the parent about pneumococcal disease

| **Question** | **Vaccinated group N=162** | | | **Unvaccinated group N=38** | | |
| --- | --- | --- | --- | --- | --- | --- |
|  | **Yes**  **n(%)** | **No**  **n(%)** | **Don’t know n(%)** | **Yes**  **n(%)** | **No**  **n(%)** | **Don’t know n(%)** |
| Have you heard about pneumococcal disease?* | 133 (82.1) | 29 (17.9) | - | 13 (34.2) | 25 (65.8) | - |
| Are babies at risk of developing pneumococcal disease? | 108 (81.2)** | 7 (5.3) | 18 (13.5) | 8 (61.5) | 1 (7.7) | 4 (30.8) |
| Are children >1 year of age at risk of developing pneumococcal disease? | 90 (67.7) | 9 (6.8) | 34 (25.6) | 9 (69.2) | 0 | 4 (30.8)) |
| Are adults at risk of developing pneumococcal disease? | 40 (30.1) | 31 (23.3) | 62 (46.6) | 4 (30.8) | 4 (30.8) | 5 (38.5) |
| Are adults >65 years of age at risk of developing pneumococcal disease? | 34 (25.6) | 19 (14.3) | 80 (60.2) | 5 (38.5) | 2 (15.4) | 6 (46.2) |
| Can pneumococcal disease include ear infections? | 40 (30.1) | 14 (10.5) | 79 (59.7) | 4 (30.8) | 1 (7.7) | 8 (61.5) |
| Can pneumococcal disease include lung infection / pneumonia? | 94 (70.7) | 4 (3.0) | 35 (26.3) | 9 (69.2) | 2 (15.4) | 2 (15.4) |
| Can pneumococcal disease include blood infections? | 41 (30.8) | 22 (16.5) | 70 (52.6) | 6 (46.2) | 2 (15.4) | 5 (38.5) |
| Can pneumococcal disease include brain infections? | 68 (51.1) | 11 (8.3) | 54 (40.6) | 9 (69.2) | 2 (15.4) | 2 (15.4) |
| Can bacteria cause pneumococcal disease? | 83 (62.4) | 10 (7.5) | 40 (30.1) | 7 (53.9) | 1 (7.7) | 5 (38.5) |
| Can virus cause pneumococcal disease? | 92 (69.2) | 8 (6.0) | 33 (24.8) | 6 (46.2) | 1 (7.7) | 6 (46.2) |
| Can parasites cause pneumococcal disease? | 22 (16.5) | 32 (24.1) | 79 (59.4) | 5 (38.5) | 1 (7.7) | 7 (53.9) |
| Can traditional medicine prevent pneumococcal disease? | 7 (5.3) | 73 (54.9) | 53 (39.9) | 2 (15.4) | 4 (30.8) | 7 (53.9) |
| Can good hygiene prevent pneumococcal disease? | 93 (69.9) | 19 (14.3) | 21 (15.8) | 10 (76.9) | 1 (2.6) | 2 (15.4) |
| Can vaccination prevent pneumococcal disease? | 126 (94.7) | 7 (5.3) | 29 (21.8) | 11 (84.6) | 2 (15.4) | 2 (15.4) |

Vaccinated group = Parents whose children had received PCV or parents who intended to have their child vaccinated

Unvaccinated group = Parents whose children had not received PCV or parents who had no intention of having their child vaccinated

N = total number of parents, n (%) = number (percentage) of parents in a given category

*Subsequent responses do not include 29 subjects in the Vaccinated group and 25 in the Unvaccinated group who had not heard about pneumococcal disease

**Percentage of parents who knew about pneumococcal disease
