# Supplementary material for: Parental knowledge, attitudes and perception of pneumococcal disease and pneumococcal conjugate vaccines in Singapore: a questionnaire-based assessment
Source: BMC Public Health. 2016 Sep 2;16(1):923. doi: 10.1186/s12889-016-3597-5 (PMC5010741; doi:10.1186/s12889-016-3597-5)
Supplement: Additional file 3: — Table S3. Knowledge of parent about pneumococcal conjugate vaccine. (DOCX 36 kb) [file 12889_2016_3597_MOESM3_ESM.docx]

**Table S3:** Knowledge of parent about pneumococcal conjugate vaccine

| **Question** | **Response** | **Vaccinated group  N = 162**  **n (%)** | **Unvaccinated group  N = 38**  **n (%)** |
| --- | --- | --- | --- |
| Have you heard about PCV before? | No | 50 (30.9) | 24 (63.2) |
|  | Yes | 112 (69.1) | 14 (36.8) |
| From where did you hear about PCV*? | Healthcare Professional (doctor or nurse) | 97 (59.9) | 12 (31.6) |
|  | Media publicity such as TV, radio, newspapers, magazines | 7 (4.3) | 0 |
|  | Friend or family members | 8 (4.9) | 1 (2.6) |
|  | Immunisation brochures, leaflets or posters in the doctor’s clinics | 22 (13.6) | 5 (13.2) |
|  | The internet | 2 (1.2) | 1 (2.6) |
|  | Other | 2 (1.2) | 0 |
|  | NA | 50 (-) | 24 (-) |
| Which of the following sources of information would you consider most influential to your decision making regarding vaccination with PCV*? | Healthcare Professional (doctor or nurse) | 151 (93.2) | 33 (86.8) |
|  | Media publicity such as TV, radio, newspapers, magazines | 40 (24.7) | 9 (23.7) |
|  | Friend or family members | 51 (31.5) | 13 (34.2) |
|  | Immunisation brochures, leaflets or posters in the doctor’s clinics | 78 (48.1) | 19 (50.0) |
|  | The internet | 22 (13.6) | 13 (34.2) |
|  | Other | 3 (1.9) | 0) |
| The PCV is included in the National Childhood immunisation Program in Singapore | Yes | 92 (56.8) | 13 (34.2) |
|  | No | 29 (17.9) | 6 (15.8) |
|  | Do not know | 41 (25.3) | 19 (50.0) |

Vaccinated group = Parents whose children had received PCV or parents who intended to have their child vaccinated

Unvaccinated group = Parents whose children had not received PCV or parents who had no intention of having their child vaccinated

N = total number of parents, n (%) = number (percentage) of parents in a given category,

PCV = pneumococcal conjugate vaccine

*Note: Since a parent can choose more than one option, the percentages will not add up to 100%.

NA = Not applicable (Parents who had not heard about pneumococcal conjugate vaccine)
