# Supplementary material for: Parental knowledge, attitudes and perception of pneumococcal disease and pneumococcal conjugate vaccines in Singapore: a questionnaire-based assessment
Source: BMC Public Health. 2016 Sep 2;16(1):923. doi: 10.1186/s12889-016-3597-5 (PMC5010741; doi:10.1186/s12889-016-3597-5)
Supplement: Additional file 4: — Table S4. Parental intent to vaccinate with PCV. (DOCX 37 kb) [file 12889_2016_3597_MOESM4_ESM.docx]

Table S4: Parental intent to vaccinate with PCV

|  | | **Vaccinated group  N = 162**  **n (%)** | **Unvaccinated group**  **N = 38**  **n (%)** |
| --- | --- | --- | --- |
| Who recommended that your child receive the PCV?^#^ | Doctor | 51 (31.5) | 6 (15.8) |
|  | Nurse | 88 (54.3) | 9 (23.7) |
|  | Friends or Family | 20 (12.3) | 2 (5.3) |
|  | Child care/day care | 2 (1.2) | 0 |
|  | No one recommended | 33 (20.4) | 24 (63.2) |
| Knowing the PCV is included in the National Childhood Immunisation Programme in Singapore. Would you be willing for your child to be vaccinated with the PCV? | No | 2 (1.2) | 13 (34.2) |
|  | Yes | 160 (98.8) | 25 (65.8) |
| If the PCV was free of charge, would you be willing for your child to be vaccinated with the vaccine? | No | 1 (0.6) | 9 (23.7) |
|  | Yes | 161 (99.4) | 29 (76.3) |
| If you had to pay for the PCV, would you be willing for your child to be vaccinated with the vaccine? | No | 9 (5.6) | 19 (50.0) |
|  | Yes | 153 (94.4) | 19 (50.0) |
| How much are you willing to pay for your child to be vaccinated with the PCV? | < 50 SGD per jab | 73 (47.7) | 13 (68.4) |
|  | 50 to 100 SGD per jab | 55 (35.9) | 6 (31.6) |
|  | 101 to 150 SGD per jab | 25 (16.3) | 0 |
|  | NA* | 9 | 19 |
| Do you intend to let your child receive the PCV? | Yes | 76 (46.9) | 0 |
|  | No | 0 | 38 (100) |
|  | N.A** | 86 | 0 |

Vaccinated group = Parents whose children had received PCV or parents who intended to have their child vaccinated

Unvaccinated group = Parents whose children had not received PCV or parents who had no intention of having their child vaccinated

N = total number of parents, n (%) = number (percentage) of parents in a given category, PCV = pneumococcal conjugate vaccine, SGD = Singapore Dollar

NA* = Not applicable (i.e. parents who were not interested to pay from their pocket)

NA** = Not applicable (i.e. parents whose children had already received PCV at the time of study entry)

#Note: Since a parent can choose more than one option, the percentages will not add up to 100%
