# Supplementary material for: Parental knowledge, attitudes and perception of pneumococcal disease and pneumococcal conjugate vaccines in Singapore: a questionnaire-based assessment
Source: BMC Public Health. 2016 Sep 2;16(1):923. doi: 10.1186/s12889-016-3597-5 (PMC5010741; doi:10.1186/s12889-016-3597-5)
Supplement: Additional file 5: — Table S5. Polychoric correlation coefficients between items for perceived benefits and barriers of PCV (15 questions ordinal using 5-point Likert scale). (DOCX 38 kb) [file 12889_2016_3597_MOESM5_ESM.docx]

Table S5 Polychoric correlation coefficients between items for perceived benefits and barriers of PCV (15 questions ordinal using 5-point Likert scale)

| **TYPE** | **ITEMS** | **Q1** | **Q2** | **Q3** | **Q4** | **Q5** | **Q6** | **Q7** | **Q8** | **Q9** | **Q10** | **Q11** | **Q12** | **Q13** | **Q14** | **Q15** |
| --- | --- | --- | --- | --- | --- | --- | --- | --- | --- | --- | --- | --- | --- | --- | --- | --- |
| N | . | 200 | 200 | 200 | 200 | 200 | 200 | 200 | 200 | 200 | 200 | 200 | 200 | 200 | 200 | 200 |
| MEAN | . | 4.29 | 4.08 | 4.44 | 2.59 | 1.94 | 3.48 | 2.83 | 3.16 | 3.80 | 3.61 | 3.42 | 2.17 | 2.51 | 2.97 | 3.63 |
| STD | . | 0.71 | 0.81 | 0.68 | 1.22 | 1.08 | 0.69 | 0.76 | 0.72 | 1.09 | 0.96 | 1.12 | 0.86 | 0.84 | 0.97 | 1.10 |
| Correlation coefficient | Q1 | 1.00 | . | . | . | . | . | . | . | . | . | . | . | . | . | . |
|  | Q2 | **0.74** | 1.00 | . | . | . | . | . | . | . | . | . | . | . | . | . |
|  | Q3 | **0.67** | **0.64** | 1.00 | . | . | . | . | . | . | . | . | . | . | . | . |
|  | Q4 | 0.03 | 0.02 | 0.05 | 1.00 | . | . | . | . | . | . | . | . | . | . | . |
|  | Q5 | -.18 | -.18 | -.20 | **0.46** | 1.00 | . | . | . | . | . | . | . | . | . | . |
|  | Q6 | **0.38** | **0.45** | **0.45** | 0.15 | -.05 | 1.00 | . | . | . | . | . | . | . | . | . |
|  | Q7 | -.18 | -.17 | -.23 | 0.01 | 0.20 | -.15 | 1.00 | . | . | . | . | . | . | . | . |
|  | Q8 | 0.08 | 0.03 | 0.03 | 0.06 | 0.19 | -.10 | 0.16 | 1.00 | . | . | . | . | . | . | . |
|  | Q9 | -.10 | -.04 | -.16 | 0.12 | 0.01 | -.19 | 0.16 | 0.20 | 1.00 | . | . | . | . | . | . |
|  | Q10 | -.12 | -.13 | -.17 | 0.10 | 0.08 | -.08 | 0.14 | 0.29 | **0.44** | 1.00 | . | . | . | . | . |
|  | Q11 | -.17 | -.10 | -.12 | 0.22 | 0.20 | 0.06 | 0.08 | 0.08 | **0.35** | **0.50** | 1.00 | . | . | . | . |
|  | Q12 | -.51 | -.54 | -.61 | 0.15 | 0.14 | -.37 | 0.19 | 0.06 | 0.11 | 0.14 | 0.07 | 1.00 | . | . | . |
|  | Q13 | -.30 | -.28 | -.17 | 0.20 | 0.22 | -.27 | 0.25 | 0.18 | 0.11 | 0.28 | 0.03 | 0.25 | 1.00 | . | . |
|  | Q14 | -.31 | -.35 | -.07 | 0.17 | 0.17 | -.12 | 0.21 | 0.04 | **0.31** | 0.25 | 0.19 | 0.13 | **0.37** | 1.00 | . |
|  | Q15 | -.18 | -.28 | -.10 | 0.29 | 0.10 | -.17 | 0.25 | 0.15 | **0.32** | **0.53** | **0.32** | 0.23 | **0.34** | **0.42** | 1.00 |

Q = Question – see Supplementary table 1 for the list of individual questions

N = total number of subjects

STD = Standard Deviation

Shading/bold indicates a moderate to very strong positive correlation or relationship between items

The following numerical scores were used for 5-point Likert scale.

Strongly agree - 1

Moderately agree - 2

Neutral - 3

Moderately disagree - 4

Strongly disagree - 5
